# Fenestration of the posterior cerebral artery P1-P2 junction: A case report

**DOI:** 10.1016/j.radcr.2024.02.088

**Published:** 2024-03-14

**Authors:** Hideki Endo, Hirohiko Nakamura

**Affiliations:** Department of Neurosurgery, Nakamura Memorial Hospital, South 1, West 14, Chuo-ku, Sapporo, Hokkaido 060-8570, Japan

**Keywords:** Anatomical variation, Fenestration, Magnetic resonance angiography, Magnetic resonance imaging, Posterior cerebral artery, P1-P2 junction

## Abstract

Fenestration is a rare anatomical variation in the posterior cerebral artery. To the best of our knowledge, there are few reports of fenestrations at the posterior cerebral artery P1-P2 junction. Herein, we report a case of fenestration of the posterior cerebral artery P1-P2 junction diagnosed by 3-T magnetic resonance imaging and magnetic resonance angiography. A 75-year-old woman visited our hospital because of headaches. Magnetic resonance imaging incidentally showed fenestration around the P1-P2 segment of the right posterior cerebral artery. Magnetic resonance angiography revealed a small fenestration at the right posterior cerebral artery P1-P2 junction. The vessel diameter of both limbs forming the fenestration was nearly equal. Careful imaging assessment is important to identify fenestration of the posterior cerebral artery P1-P2 junction. Both magnetic resonance angiography and magnetic resonance imaging were useful for diagnosis in this case.

## Introduction

Fenestration is an anatomical variation in which a single vessel divides into 2 channels, each with endothelial and muscular layers, which unite distally into a single lumen [1]. Fenestration may alter microvascular flow dynamics that increase the risk of aneurysm formation and may also be involved in any pathological vascular manifestations [1]. The P1 segment of the posterior cerebral artery (PCA) refers to the segment from the basilar artery bifurcation to its confluence with the posterior communicating artery (PCoA), and the P2 segment refers to the segment after confluence with the PCoA [2]. Fenestration is rare among anatomical variants in the PCA, and in particular, to our knowledge, there are only a few reports of fenestrations at the P1-P2 junction [3,4]. Herein, we report a case of fenestration at the PCA P1-P2 junction diagnosed by 3-T magnetic resonance imaging (MRI) and magnetic resonance angiography (MRA).

## Case report

**Fig. 1:**
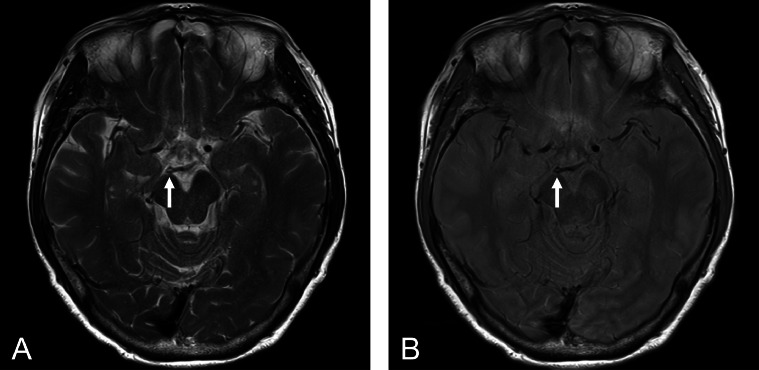
Magnetic resonance imaging (A, T2-weighted image; B, proton density-weighted image) showing fenestration around the P1-P2 segment of the right posterior cerebral artery (arrows).

**Fig. 2:**
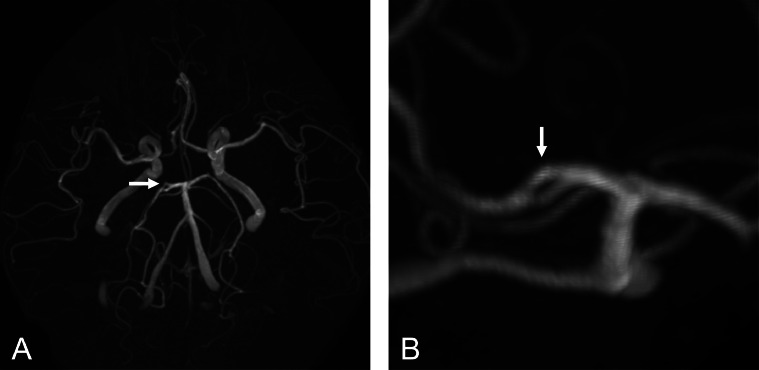
Magnetic resonance angiography (A, anteroposterior view; B, oblique view) showing fenestration at the P1-P2 junction of the right posterior cerebral artery (arrows).

A 75-year-old woman with a history of allergic rhinitis and sleep apnea syndrome visited our hospital complaining of 2 episodes of nighttime headaches. The headaches were not a new symptom for her; they were not severe, but rarely occurred at night. She did not have any prior imaging studies. At the time of the visit, she was asymptomatic, and there were no apparent abnormal neurological findings. She underwent MRI/MRA (3-T Magnetom Vida; Siemens, Erlangen, Germany), which showed chronic sinusitis but no organic lesions associated with possible headache. MRI (T2-weighted and proton density-weighted images) incidentally showed fenestration around the P1-P2 segment of the right PCA (Fig. 1). MRA revealed a small fenestration at the right PCA P1-P2 junction (Fig. 2). The vessel diameter in each limb forming the fenestration was almost equal. There was no obvious concomitant vascular lesion, and we considered the patient's headaches were not related to the fenestration. We could not rule out the possibility that sleep apnea may have played a role in her symptoms, but she had only two episodes and we decided to explore if they persisted. The patient's headaches were treated with oral medications (non-steroidal anti-inflammatory drugs).

## Discussion

In this study, we presented a rare case of fenestration at the PCA P1-P2 junction. Aplasia or hypoplasia of the P1 segment and fetal-type PCoA are commonly encountered clinically, but other anatomical variations in the PCA are rare, with a prevalence of 1.28% [3,5,6]. In a previous study of anatomical variations of the PCA in 2350 patients on 3-T MRA, fenestration was identified in 8 patients (0.34%), early bifurcation in 8 patients (0.34%), complete duplication in 1 patient (0.04%), and hyperplastic anterior choroidal artery in 13 patients (0.55%) [3]. In a previous study involving cadavers, PCA fenestration was detected in only 4 of 468 (0.85%) (200 fetal and 268 adult) cadavers [7]. In other dissection studies, PCA fenestrations were found at the P1 segment in 1 of 100 brains and at the P2 segment in 1 of 37 brains [8,9].

Fenestration was observed at the PCA P1-P2 junction in our patient (Figs. 1 and 2). To the best of our knowledge, only a few reports have demonstrated fenestrations at the P1-P2 junction [3,4]. In the MRI study described above, fenestration at the P1-P2 junction was observed in only 2 of 2350 (0.09%) patients [3]. In the dissection study, only 1 case with fenestration at the P1-P2 junction was reported [4]. Therefore, fenestration at the PCA P1-P2 junction is extremely rare. The present report provides additional evidence that fenestration can occur at the P1-P2 junction. Although the clinical significance of this rare anatomical variation may be limited, knowledge of this variation may provide useful information for preoperative evaluation around the PCA.

The significance of fenestration in the context of neurovascular pathologies is controversial. Fenestration may alter microvascular flow dynamics, and the hemodynamic changes may not only predispose to aneurysm formation, but also to dissection and/or thromboembolism [1]. We considered that such concomitant vascular pathologies could cause headaches in patients with fenestrations. However, fenestration in this case was not accompanied by vascular lesions, and we determined that there was no association with headache.

In the present case, fenestration at the PCA P1-P2 segment was identified by both MRA and MRI (Figs. 1 and 2). The ability to evaluate the PCA P1-P2 segment by MRI as well as MRA may be useful for the following reasons: first, in the assessment of this rare anatomical variation, it may be feasible to first detect the variation on MRI and later investigate in detail using MRA. Second, MRA can evaluate only the lumen of the vessel, whereas MRI can also evaluate the area outside the lumen. Careful imaging assessment is important to identify fenestration at the P1-P2 junction. Both MRA and MRI were useful for diagnosis in this case.

## Conclusions

We describe a rare case of fenestration of the PCA P1-P2 junction diagnosed by 3-T MRI/MRA. Careful image assessment is important to identify this rare anatomical variation.

## Ethical statement

All procedures performed in studies involving human participants were in accordance with the ethical standards of the institutional and/or national research committee and with the 1964 Helsinki Declaration and its later amendments or comparable ethical standards. The study was approved by the Ethics Committee of Nakamura Memorial Hospital (No. 2023102901).

## Patient consent

This study was approved by the institutional review board, and informed consent was obtained from the patient.

## CRediT authorship contribution statement

**Hideki Endo:** Conceptualization, Methodology, Validation, Formal analysis, Investigation, Resources, Data curation, Writing – original draft, Writing – review & editing, Visualization, Project administration. **Hirohiko Nakamura:** Supervision.
